# Targeting Cathepsins in Neurodegeneration: Biochemical Advances

**DOI:** 10.3390/biomedicines13123019

**Published:** 2025-12-09

**Authors:** Francesca Di Matteo, Mariapia Vietri, Simone D’Alessio, Tania Ciaglia, Erica Federica Vestuto, Giacomo Pepe, Ornella Moltedo, Veronica Di Sarno, Simona Musella, Carmine Ostacolo, Fabio Cominelli, Pietro Campiglia, Alessia Bertamino, Maria Rosaria Miranda, Vincenzo Vestuto

**Affiliations:** 1 Department of Pharmacy, University of Salerno, Via G. Paolo II, 84084 Fisciano, Italy; fdimatteo@unisa.it (F.D.M.); mvietri@unisa.it (M.V.); s.dalessio10@studenti.unisa.it (S.D.); tciaglia@unisa.it (T.C.); gipepe@unisa.it (G.P.); moltedo@unisa.it (O.M.); vdisarno@unisa.it (V.D.S.); smusella@unisa.it (S.M.); costacolo@unisa.it (C.O.); pcampiglia@unisa.it (P.C.); abertamino@unisa.it (A.B.); 2 Department of Neurology, Sant’Antonio Abate Hospital, Via Eusebio Pastori 4, 21013 Gallarate, Italy; erica.vestuto@asst-valleolona.it; 3 National Biodiversity Future Center (NBFC), 90133 Palermo, Italy; 4 Department of Medicine, Digestive Health Research Institute, Case Western Reserve University School of Medicine, Cleveland, OH 44106, USA; fabio.cominelli@uhhospitals.org

**Keywords:** cathepsins, cathepsin inhibitors, neurodegeneration, neurodegenerative disease, ER stress, mitochondrial disfunction, biochemical pathways, drug development

## Abstract

**Background/Objectives**: Cathepsins, lysosomal proteases crucial for neuronal proteostasis, mediate the clearance of misfolded and aggregated proteins. Their dysregulation is implicated in neurodegenerative and neuropsychiatric disorders such as Alzheimer’s, Parkinson’s, and Huntington’s diseases. These conditions are characterized by toxic protein accumulation and impaired clearance, which exacerbate cellular stress responses, including the unfolded protein response (UPR), oxidative damage, and mitochondrial dysfunction. This review aims to summarize current knowledge on cathepsin roles in these pathways and assess their therapeutic potential. **Methods**: A comprehensive literature review was conducted, focusing on recent in vitro and in vivo studies investigating cathepsin function, inhibition, and modulation. Mechanistic insights and pharmacological approaches targeting cathepsins were analyzed, with attention to challenges in translating preclinical findings to clinical settings. **Results**: Cathepsins demonstrate a dual role: their proteolytic activity supports neuronal health by degrading toxic aggregates, but altered or insufficient activity may worsen proteotoxic stress. Studies reveal that cathepsins regulate autophagy, apoptosis, and neuroinflammation both intracellularly and extracellularly. Despite promising mechanistic data, clinical translation is hindered by issues such as poor inhibitor selectivity, limited brain penetration, and variability across preclinical models. **Conclusions**: Targeting cathepsins presents a promising strategy for treating neurodegenerative and neuropsychiatric disorders, but significant challenges remain. Future research should focus on improving drug specificity and delivery, and on developing standardized models to better predict clinical outcomes.

## 1. The Lysosomal System and Its Role in Proteostasis and Neuronal Health

The lysosome is a membrane-bound organelle characterized by a highly acidic lumen (pH ~4.5–5.0) and containing more than 60 acid hydrolases, including proteases, glycosidases, lipases, and nucleases, that enable the degradation of proteins, carbohydrates, lipids, and nucleic acids. Beyond its canonical role as the cellular “waste disposal system”, the lysosome is involved in several essential physiological processes, including energy homeostasis, signal transduction, plasma membrane repair, and innate immunity.

Autophagy, a fundamental mechanism for maintaining cellular proteostasis, directs intracellular cargo toward lysosomal degradation through three main pathways [1,2]:

1. Macroautophagy, the most studied form, involves the formation of double-membraned autophagosomes that sequester damaged organelles or protein aggregates and subsequently fuse with lysosomes to form autolysosomes where degradation occurs.
2. Microautophagy, in which invaginations of the lysosomal membrane directly engulf cytoplasmic content.
3. Chaperone-mediated autophagy (CMA), a selective process whereby cytosolic proteins containing the KFERQ motif are recognized by HSPA8 and translocated into the lysosome via the LAMP-2A receptor.

All three forms converge at the lysosome, where their substrates are broken down into basic biomolecules, amino acids, monosaccharides, and fatty acids, and recycled back into the cytoplasm for reuse [3]. Proper functioning of the autophagy-lysosome system is crucial for neuronal health, as it prevents the accumulation of misfolded proteins and dysfunctional organelles: features that are hallmark pathological traits of many neurodegenerative diseases [4].

A key group of lysosomal enzymes is the cathepsins, proteases that are optimally active in acidic environments and are responsible for protein turnover, antigen presentation, tissue remodeling, cell death regulation, and extracellular signaling [5,6].

## 2. The Role of Cathepsins in Neurons and Their Alteration Under Stress

Cathepsins are a family of proteolytic enzymes primarily localized in lysosomes, classified according to the nature of the catalytic residue in their active sites into three main classes: cysteine (including CTSB, CTSL, CTSC, CTSK, CTSH, CTSZ/X), aspartic (CTSD and CTSE), and serine proteases (CTSA and CTSG) [5]. These enzymes are synthesized as inactive proenzymes and undergo maturation via lysosomal acidification, which ensures their activation and catalytic efficiency [7].

Within the central nervous system (CNS), cathepsins play an essential role in maintaining cellular homeostasis and proper neuronal function. They are fundamental for protein turnover, degradation of protein aggregates, antigen presentation via the MHC-II pathway, extracellular matrix remodeling, organelle maturation, and regulation of programmed cell death [8,9]. Their primary function is mediated through their contribution to autophagic activity, a critical process for the selective degradation of damaged organelles and misfolded proteins [10].

Under physiological conditions, cathepsins support synaptic plasticity, regulation of the cell cycle in neural progenitors, and synaptic remodeling in response to neuronal activity [11]. Several studies have shown that modulation of cathepsin activity is critical for neurogenesis and for regulating the neuronal microenvironment by degrading the extracellular matrix and modulating glia-neuron interactions [12].

However, a finely tuned balance is required: both insufficient and excessive cathepsin activity can lead to pathological outcomes. Loss of cathepsin function has been associated with the accumulation of toxic aggregates, lysosomal dysfunction, and neuronal death [13]. Conversely, uncontrolled release of cathepsins into the cytosol following lysosomal membrane permeabilization can activate apoptotic or inflammatory cascades with neurotoxic consequences [14].

In summary, cathepsins play a dual role in the CNS: a homeostatic and protective function under physiological conditions, and a potentially pathogenic role when their regulation is disrupted by stress stimuli or structural damage. This delicate balance makes them a critical node in the cellular mechanisms regulating neuronal survival and vulnerability to neurodegeneration.

### Stress-Induced Dysregulation

The interplay between endoplasmic reticulum (ER) stress, oxidative damage, and lysosomal dysfunction represents a central node in neurodegenerative pathology. These processes are not isolated; they potentiate each other and converge on proteostasis disruption, as also detailed in mechanistic studies from oncology and neurobiology models [15,16,17].

- **ER Stress**

In neurodegenerative diseases such as Alzheimer’s disease (AD) and Parkinson’s disease (PD), the persistent accumulation of misfolded proteins, such as amyloid-β (Aβ), α-synuclein (αSyn), and mutant huntingtin (mHtt), overwhelms the folding capacity of the endoplasmic reticulum (ER), triggering a protective signaling network known as the unfolded protein response (UPR) [18]. The UPR is mediated by three main ER stress sensors: protein kinase RNA-like endoplasmic reticulum kinase (PERK), inositol-requiring enzyme 1 (IRE1), and activating transcription factor 6 (ATF6). Upon ER stress, the dissociation of the chaperone immunoglobulin heavy-chain-binding protein (BiP/GRP78) from these sensors leads to their activation. PERK phosphorylates eukaryotic initiation factor 2 alpha (eIF2α), attenuating global protein synthesis; IRE1 splices X-box binding protein 1 (XBP1) mRNA, increasing expression of folding and degradation-related genes; ATF6 translocates to the Golgi and is cleaved into an active transcription factor [19].

In neurons, the high basal metabolic demand and post-mitotic state render them particularly vulnerable to chronic ER stress. In vitro studies on primary neuronal cultures have shown that prolonged ER stress induces the formation of markedly enlarged autophagic vesicles and so-called “giant lysosomes”. These structures reflect a compensatory upregulation of lysosomal biogenesis and flux, aimed at managing the burden of misfolded proteins through enhanced autophagic clearance [20,21]. This phenomenon suggests a cellular attempt to reinforce the degradative capacity of the endolysosomal system. However, sustained ER stress also impairs lysosomal acidification and cathepsin maturation, ultimately reducing proteolytic efficiency and promoting proteotoxic stress.

Experimental models have demonstrated that prolonged activation of the UPR contributes to synaptic loss and neurodegeneration by upregulating pro-apoptotic factors such as C/EBP homologous protein (CHOP), inducing mitochondrial dysfunction, and inhibiting global protein synthesis [22]. Collectively, this evidence supports the concept that while transient ER stress responses are neuroprotective, chronic ER stress disrupts lysosomal homeostasis and cathepsin activity, facilitating the accumulation of neurotoxic aggregates and contributing to disease progression [23].

- **Oxidative Stress**

Oxidative stress, a major driver of neuronal injury in AD and PD, arises from excessive production of reactive oxygen species (ROS), particularly in dysfunctional mitochondria or activated immune cells. ROS can oxidatively modify lipids, nucleic acids, and proteins, including lysosomal proteins and cysteine proteases like CTSB and cathepsin L (CTSL). Oxidation of the active-site cysteine by ROS modifies the thiol group, forming sulfenic, sulfinic, or sulfonic acid derivatives. This chemical modification disrupts the nucleophilic properties of the catalytic cysteine, thereby impairing the enzyme’s ability to cleave peptide bonds and reducing its catalytic efficiency. In addition, lipid peroxidation of the lysosomal membrane promotes lysosomal membrane permeabilization (LMP), facilitating the cytosolic release of cathepsins and triggering downstream caspase-dependent and caspase-independent cell death pathways. Studies in neuronal cells and animal models show that such redox-mediated impairments in lysosomal integrity aggravate protein accumulation and neurodegeneration [24].

Recent findings show that ROS-driven lysosomal dysfunction can be counteracted by activating nuclear factor erythroid 2-related factor 2 (NRF2) and transcription factor EB (TFEB) [25]. NRF2 enhances the expression of antioxidant genes, while TFEB controls lysosomal biogenesis and autophagy. Their coordinated activation has been shown to restore lysosomal acidification, stabilize cathepsins, and reduce proteotoxicity in cellular models of neurodegeneration [26].

- **Mitochondrial dysfunction**

Mitochondrial dysfunction plays a central role in the pathophysiology of neurodegenerative diseases. Defective mitophagy, often due to mutations in PTEN-induced putative kinase 1 (PINK1) or leucine-rich repeat kinase 2 (LRRK2), leads to the accumulation of damaged mitochondria and elevated ROS production. Inadequate ATP generation impairs the function of vacuolar-type H^+^-ATPases (V-ATPases), responsible for lysosomal acidification and activation of cathepsins. In PD models, failure of mitochondrial clearance leads to the accumulation of dysfunctional organelles, disrupted autophagic flux, and further impairment of the lysosomal system [27].

- **ER Mitochondria Crosstalk and Neuroinflammation**

Mitochondria-associated membranes (MAMs), the physical contact sites between the endoplasmic reticulum (ER) and mitochondria, play a central role in regulating intracellular calcium (Ca^2+^) flux, lipid metabolism, and apoptotic signaling [28]. Disruption of MAM integrity, observed in both Alzheimer’s and Parkinson’s disease, alters Ca^2+^ homeostasis and impairs ER-mitochondria communication, leading to mitochondrial Ca^2+^ overload and increased oxidative stress. This imbalance contributes to mitochondrial dysfunction and disturbs lysosomal Ca^2+^-dependent mechanisms that control acidification and enzyme activation, including cathepsin processing [29].

In parallel, chronic neuroinflammation exacerbates proteostasis failure. Microglia and astrocytes, when activated by stimuli such as Aβ, α-synuclein, or pro-inflammatory cytokines, upregulate the expression and secretion of lysosomal enzymes, particularly cathepsin B (CTSB), into the extracellular environment. Extracellular CTSB plays a non-canonical role by cleaving extracellular matrix components and activating innate immune pathways. Specifically, extracellular CTSB has been shown to trigger activation of the NLRP3 inflammasome in microglia and peripheral macrophages, leading to cleavage of pro-caspase-1 and release of interleukin-1β (IL-1β), a key mediator of neuroinflammation and neuronal damage [30].

Moreover, under oxidative or proteotoxic stress, LMP leads to the leakage of cathepsins, especially CTSB, CTSL, and CTSD, into the cytosol. Once in the cytoplasm, these proteases can directly activate apoptotic pathways: CTSB and CTSL cleave pro-caspases 3 and 9 or degrade the anti-apoptotic protein Bcl-2. At the same time, cathepsin D can enhance mitochondrial outer membrane permeabilization by cleaving Bid and activating the Bax/Bak pathway. The consequence is the amplification of both caspase-dependent apoptosis and caspase-independent forms of cell death, such as lysosome-dependent necrosis or ferroptosis. Notably, the combination of extracellular CTSB-induced inflammasome activation and intracellular cathepsin-driven apoptosis promotes a chronic inflammatory environment that sustains glial activation and neurotoxicity. This creates a feed-forward loop in which neuroinflammation and lysosomal dysfunction reinforce each other, accelerating neuronal loss and disease progression in AD and PD [21,27].

Therefore, the interplay between oxidative stress, ER stress, mitochondrial dysfunction, and cathepsin dysregulation is a key factor in the pathogenesis of neurodegenerative disease. The accumulation of misfolded proteins induces ER stress and activates UPR, which aims to restore proteostasis but, if chronically activated, can cause cellular dysfunction and death. Oxidative stress and consequent mitochondrial damage worsen the condition by harming cellular components and increasing protein misfolding.

**Figure 1:**
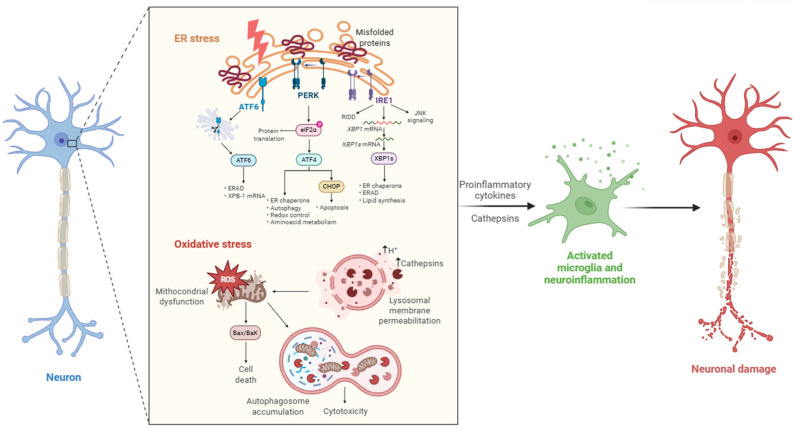
Interplay between endoplasmic reticulum stress, oxidative stress, mitochondrial dysfunction, and cathepsin dysregulation in neuronal degeneration.

The dysregulated activity of cathepsins contributes to the accumulation of toxic proteins, exacerbates ER and oxidative stress, and promotes neuroinflammation by activating pro-inflammatory pathways. Additionally, cathepsin dysregulation impairs autophagy, a vital process for removing damaged proteins and organelles, creating a vicious cycle that accelerates neurodegeneration (Figure 1).

In summary, the interplay between ER stress, oxidative stress, mitochondrial dysfunction, and cathepsin dysregulation forms a pathological feedback loop particularly relevant in neurodegenerative diseases. Impairments in unfolded protein response signaling, redox homeostasis, and mitochondrial quality control converge on the lysosomal system, altering the expression, maturation, and localization of cathepsins. The resulting autophagic failure, neuroinflammation, and cell death drive progressive neurodegeneration.

Under neurodegenerative conditions such as AD and PD, the accumulation of misfolded proteins disrupts neuronal proteostasis and leads to chronic activation of UPR. When sustained over time, UPR signaling contributes to impaired lysosomal acidification and defective cathepsin maturation, ultimately exacerbating inflammatory and degenerative processes. Oxidative stress, primarily induced by mitochondrial dysfunction, leads to excess production of ROS and lipid peroxidation, resulting in lysosomal membrane permeabilization (LMP) and inactivation of cysteine proteases such as cathepsin B (CTSB), cathepsin L (CTSL), and cathepsin D (CTSD). The massive release of cathepsins into the cytosol activates apoptotic pathways, while their extracellular release by activated microglia contributes to the secretion of proinflammatory cytokines and the amplification of neuroinflammation.

Mitochondrial dysfunction and disruption of ER-mitochondria contact sites (mitochondria-associated membranes, MAMs) further exacerbate ROS generation, impair calcium homeostasis, and disturb cathepsin regulation. These interconnected stress responses converge on lysosomal failure and autophagic impairment, fueling a vicious cycle of inflammation, proteotoxicity, and neuronal damage [28,29].

## 3. Cathepsins Dysregulation in Neurodegenerative Diseases

Growing evidence suggests a pivotal role for lysosomes in neurodegenerative diseases by regulating aggregation-prone proteins such as α-synuclein and β-amyloid. Neurons, which are post-mitotic neurons, are more susceptible to the accumulation of cellular aggregates, making autophagy crucial for maintaining cellular homeostasis. Therefore, dysfunction of lysosomal enzymes and impaired autophagic flux can lead to the accumulation of toxic protein aggregates, oxidative damage, and neuronal cell death. Moreover, dysfunctional lysosomes may contribute to ER stress by impairing the clearance of misfolded proteins, further exacerbating cellular dysfunction and neurodegeneration.

The results of numerous recent studies have suggested that aggregation-prone proteins, such as tau, α-synuclein, polyglutamine-containing proteins, and amyloid-β, can spread to other cells and regions of the brain. The cell-to-cell propagation of protein aggregates may be the general principle underlying the progressive deterioration in neurodegenerative diseases [31].

Alterations in the activity of some cysteine cathepsins and aspartyl cathepsin D may contribute to the pathogenesis of neurodegenerative disease [32,33]. These cathepsins, widely expressed in the CNS, are implicated in various neuronal functions, including synaptic plasticity. In fact, it has been shown that various protein aggregates, implicated in neurodegeneration, are substrates of these cathepsins (Table 1).

Several in vivo studies on neurodegeneration have shown significant increases in the levels of cathepsins B, L, H, C, and X in various brain regions following excitotoxin-induced neuroinflammation, such as that caused by LPS. These studies suggest that cathepsins in activated microglia can trigger pro-apoptotic factors, leading to neuronal death [43]. Cathepsin B, which is abundant in regions such as the neocortex and hippocampus, plays a critical role in neuronal development and cell proliferation by degrading proteins, lipids, and carbohydrates. Elevated cathepsin B levels have been linked to the activation of nuclear factor κB (NF-κB) and the degradation of mitochondrial transcription factor A in aged microglia, as well as the initiation of apoptosis through the activation of pro-caspases-1 and -11 [44]. Additionally, in chromaffin cells, cathepsin B is associated with the production of neurotoxic amyloid β (Aβ) peptides [45].

Cathepsin L, which is widely distributed in the brain, particularly in neurons, astrocytes, and microglia, is involved in cell–cell communication within the CNS by participating in the biosynthesis of peptide neurotransmitters like enkephalin, dynorphin, and cholecystokinin [46]. Extracellular cathepsin L also plays a crucial role in tissue remodeling, promoting axonal growth in cortical and spinal cord neurons in vitro [47]. Furthermore, in vitro studies using BV2 cells incubated with the cathepsin L inhibitor NapSul-Ile-Trp-CHO (iCL) prior to LPS stimulation were performed by Xu et al. Following cathepsin L inhibition, the caspase 8 and NF-κB pathways were reduced, together with the expression of iNOS and COX-2. These results were confirmed by in vivo studies in cathepsin L knockout mice, indicating that cathepsin L is involved in the activation of LPS-induced neuroinflammation. On the contrary, the overexpression of cathepsin L exacerbated LPS-induced neuroinflammation through the activation of the caspase 8 and NF-κB pathways, leading to increased levels of TNFα, iNOS, and COX-2 [48].

Cathepsin H, found primarily in astrocytes and secretory vesicles associated with neuropeptides, can cleave N-terminal basic residues of (Met)enkephalin and act as an endopeptidase, metabolizing neuropeptides and bradykinin. It is present in all brain regions and in high concentrations in cerebrospinal fluid, and it plays a role in TLR3/IFN-β signaling [49,50].

Cathepsin C is expressed at lower levels in the brain, mainly in the limbic system and brainstem nuclei, and is induced in activated microglia by systemic LPS injection or stimulation with IL-1β and IL-6. Cathepsin C regulates neuronal functions, such as by inducing the production of chemokine ligand 2, which attracts inflammatory cells to damaged myelin sheaths [51].

Cathepsin S is unique among cysteine cathepsins for its ability to remain active at neutral pH, allowing it to participate in extracellular proteolytic processes. It is expressed throughout the brain and released by microglia and macrophages in response to inflammatory stimuli, degrading extracellular matrix components and facilitating microglial migration to inflammation sites in the CNS [52].

Cathepsin X (also called cathepsin Z), another cathepsin implicated in neuroinflammatory processes, is a key cysteine peptidase involved in degenerative processes during aging and neurodegenerative diseases. It is primarily found in immune cells but is also widely expressed in brain cells, including microglia, astrocytes, aged neurons, and oligodendrocytes. For instance, in vitro studies on BV2 cells have demonstrated the crucial role of cathepsin X in LPS-induced neuroinflammation and the increase in microglial cathepsin X leading to microglia-mediated neurodegeneration [53]. Cathepsin X’s proteolytic activity in neurons disrupts the neurotrophic function of γ-enolase. In activated microglia, cathepsins B and X activate NF-κB, leading to the transcription of genes encoding pro-inflammatory cytokines that drive caspase activation and pyroptosis, thus exacerbating neuroinflammation and promoting the release of reactive nitrogen and oxygen species [54]. Cathepsins L and X also contribute to the NF-κB pathway, furthering neurodegeneration [34]. The degradation of neurons is intensified by the role of cathepsins B, H, L, and S in cleaving the pro-apoptotic protein Bid, which activates Bax and Bak proteins, promoting cytochrome c release from mitochondria and triggering the caspase cascade that results in apoptosis [55].

On the other hand, pharmacological inhibition or knockdown of cathepsins may alleviate microglial-mediated neuroinflammatory responses [48].

Further confirmation was found in multiple studies where in an LPS-induced neuroinflammation model, cathepsin X inhibition mitigated neurotoxicity and striatal degeneration, supporting the notion that suppressing or knocking out cathepsin X confers neuroprotective effects [56,57].

Thus, cathepsins are primarily involved in neuroinflammation, which in turn is closely linked to synaptic dysfunction and neurodegenerative diseases [4]. In fact, during neuroinflammation, activated microglia not only secrete pro-inflammatory cytokines but also release lysosomal peptidases, including cathepsins, which further amplify neuroinflammation and self-propelled neurotoxicity, leading to neurodegeneration in a vicious circle. This process is activated by the protein aggregates, such as β-amyloid (Aβ), α-synuclein (αSyn), and mutated huntingtin (Htt) [33]. In this regard, it has been demonstrated that protein aggregates found in the brains of post-mortem patients can activate microglia, leading to neuroinflammation [58,59,60,61].

### 3.1. Mechanism of Cathepsin Inhibition and Principal Classes of Inhibitors

Cathepsins may be divided into three groups (aspartic, cysteine, and serine proteases), each characterized by a catalytic triad or dyad of amino acid residues essential for proteolytic activity.

#### 3.1.1. Aspartic Proteases (CTSD and CTSE)

**Figure 2:**
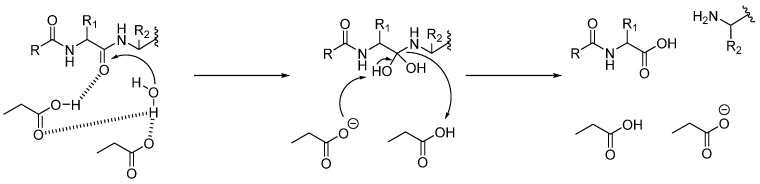
Mechanism of substrate cleavage performed by aspartic proteases.

Aspartic proteases use two aspartic acid residues in the active site to cleave the peptidic substrate. Asp33 and Asp231 are the active amino acids in CTSD, and Asp96 and Asn281 for CTSE [62,63]. One of the two aspartic acids is protonated in the enzyme, while the second one is deprotonated and acts as a general base. This one activated a water molecule, removing a proton, allowing the water to attack the substrate’s carbonyl group, forming a tetrahedral geminal diol intermediate (Figure 2). The rearrangement of this intermediate led to bond cleavage [64,65]. Aspartic protease inhibitors (such as pepstatin A or HIV-1 protease peptidomimetics) act as reversible transition-state analogues and do not use reactive warheads, thereby conferring high selectivity for aspartic proteases with negligible activity against serine or cysteine proteases.

**Figure 3:**
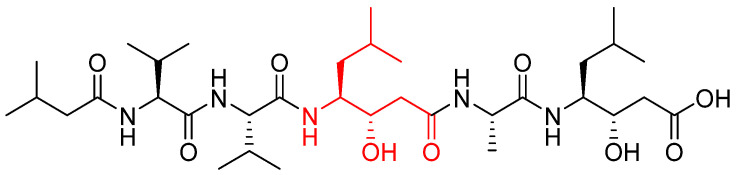
Pepstatin A structure and its portion (in red) that mimics the diol intermediate.

A well-known aspartate cathepsin inhibitor is pepstatin A, a hexapeptide containing isovaleryl-Val-Val-Sta-Ala-Sta (Figure 3); its mechanism of inhibition involves mimicking the transition state of the cleaved peptide bond, binding tightly to the enzyme’s active site, and preventing substrate interaction. In particular, pepstatin A contains the statine amino acid (Figure 3, in red), which is in turn constituted by a –CH(OH)– group that mimics the diol intermediate (shown in red in Figure 3), occupying the same position as the substrate’s carbonyl and interacting with the catalytic aspartic acids [66].

**Figure 4:**
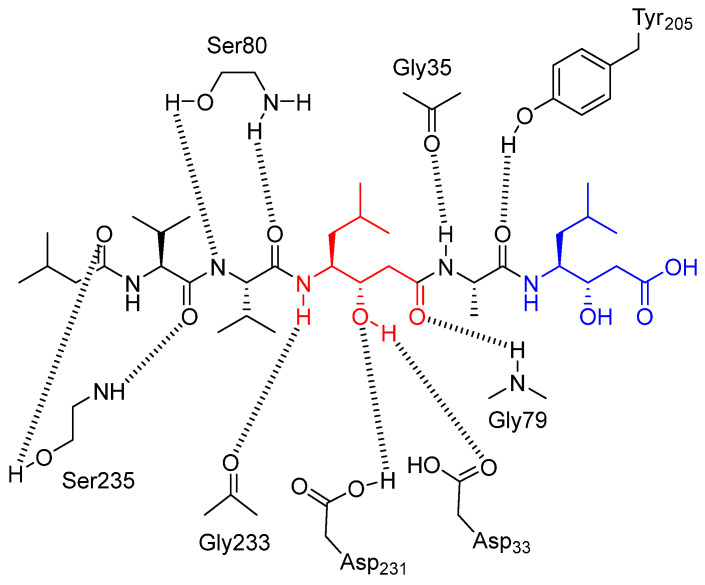
Binding mode between pepstatin A and cathepsin D. Adapted from Baldwin et al. [58].

Interactions between pepstatin A and cathepsin D have been elucidated by Baldwin et al. [62]. The complex is stabilized by numerous hydrogen bonds between backbone atoms of the inhibitor and the peptide chains of the enzyme. The central statine hydroxyl group (colored in red in Figure 4) occupies the position of a water molecule that interacts with two aspartate residues (Asp33 and Asp231) in the active site of the enzyme. In addition, the statine amino acid at the C-terminus (in blue in Figure 4) of the inhibitor makes no hydrogen bond with the enzyme, even though the presence of several donor and acceptor substituents.

**Figure 5:**
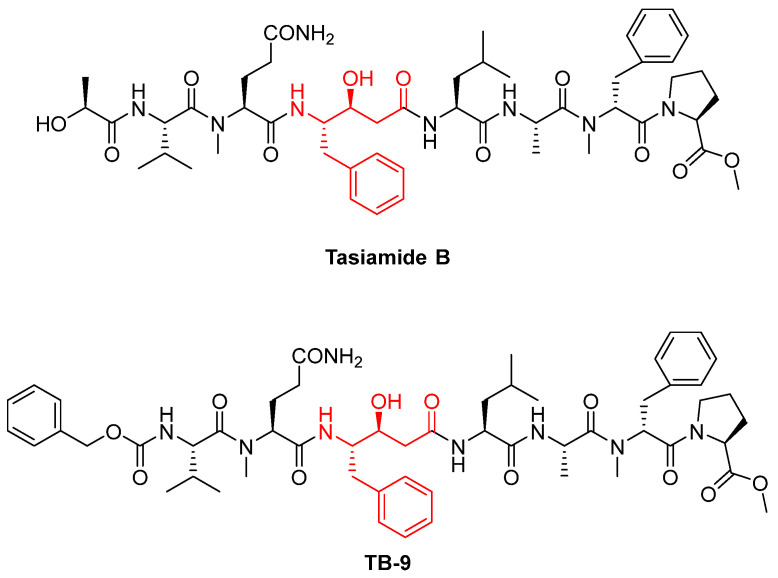
Structures of tasiamide B and its derivative TB-9.

Tasiamide B is another cathepsin D inhibitor: a linear peptide found in the marine cyanobacterium Symploca. Tasiamide B contains a hydroxyethylamine-like pharmacophore, which mimics the tetrahedral intermediate during peptide hydrolysis by cathepsin D (shown in red in Figure 5). Tasiamide has been used as a hit compound to develop other aspartic inhibitors, such as TB-9 (Figure 5), with an IC_50_ of 0.0783 nM and 0.724 nM, against CTSD and CTSE, respectively. TB-9 was obtained by truncating a lactic acid of tasiamide B and introducing a Cbz group at the N-terminus. However, although TB-9 exhibits potent inhibition, particularly against CTSD, further studies will be performed to improve cell permeability [67,68].

#### 3.1.2. Serine Proteases (CTSA and CTSG)

**Figure 6:**
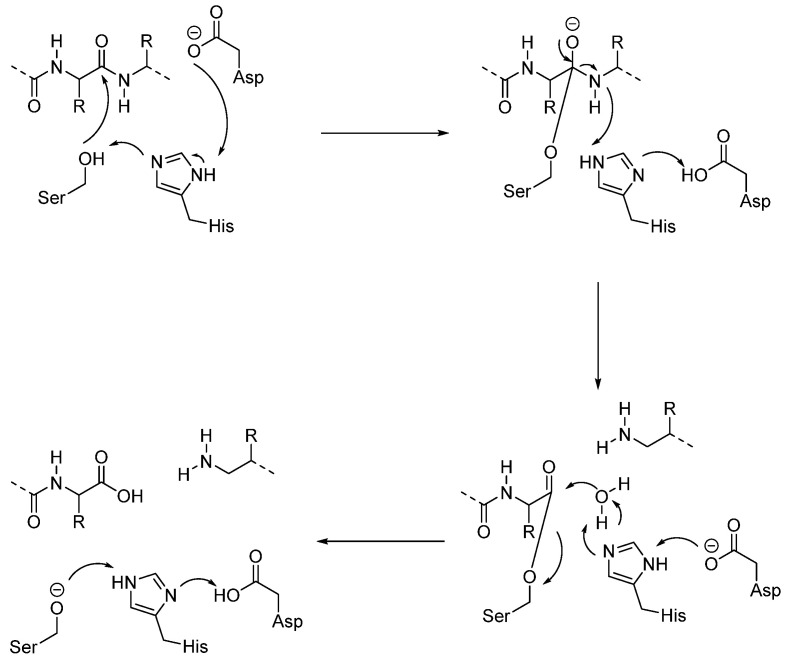
Mechanism of serine proteases cleavage.

The amino acids that form the catalytic triad of serine proteases are serine, histidine, and aspartate (Ser150, His429, Asp372 in cathepsin A, Ser195, His57, Asp102 in cathepsin G). The cleavage mechanism is similar to that of cysteine proteases (Figure 6). First, Ser attacks the carbonyl carbon of the peptide bond, forming a tetrahedral intermediate (stabilized by the oxyanion hole). At this point, the peptide bond is broken, and one product leaves. Then, His activates water to attack the acyl-enzyme, allowing the second half of the peptide to leave and regenerating the enzyme.

Serine protease inhibitors share common features with cysteine ones: for instance, an aldehyde warhead may act on both serine and cysteine proteases (such as leupeptin, described below). These compounds are therefore not categorized under this section but introduced in the cysteine protease section, consolidating all overlapping and cysteine-active warheads in one place. In contrast, epoxysuccinate inhibitors are only effective against cysteine proteases because the serine hydroxyl lacks sufficient nucleophilicity to open the epoxide ring, unlike the cysteine thiol.

#### 3.1.3. Cysteine Proteases (CTSB, CTSL, CTSC, CTSK, CTSH, CTSZ/X)

**Figure 7:**
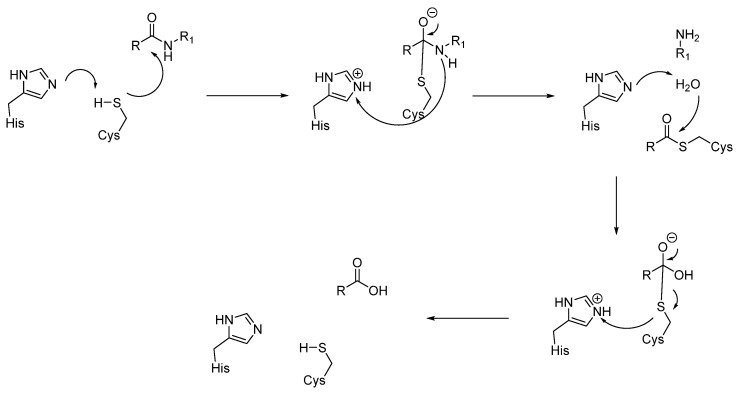
Mechanism of cleavage mediated by cysteine cathepsin proteases.

The active site of cysteine proteases is characterized by a catalytic dyad formed by a cysteine and a histidine. For instance, Cys29 and His199 form the active site of cathepsin B, while Cys25 and His159 form the active site in CTSL, or Cys234 and His381 in cathepsin C [69,70,71]. However, the mechanism of action required for proteolytic activity remains essentially the same and comprises four steps:

1. Histidine acts as a base and deprotonates the thiol from cysteine, forming the thiolate anion.
2. The thiolate attacks the carbonyl group of the substrate, giving the acyl-enzyme intermediate.
3. At this point, the tetrahedral intermediate is stabilized by an oxyanion hole. In fact, the tetrahedral intermediate is characterized by a negatively charged oxygen atom, which is stabilized by hydrogen bonds from the amide backbone, typically from NH groups of residues near the active site cleft.
4. Then, the peptide bond is cleaved, the substrate is released, and the cysteine is restored (Figure 7) [72,73].

Cysteine protease inhibition involves preventing the active cysteine from reacting. The most common form of cysteine inhibition is covalent, in which the inhibitor forms a covalent bond with the cysteine, halting the reaction with the natural substrate. The covalent inhibition could be reversible (as for the aldehyde inhibitors) or irreversible (epoxysuccinate class of inhibitors). For this reason, all inhibitors of serine and cysteine proteases are listed in the section that follows, where cross-reactivity and cysteine-selective warheads are treated together.

- **Aldehyde inhibitors**

**Figure 8:**
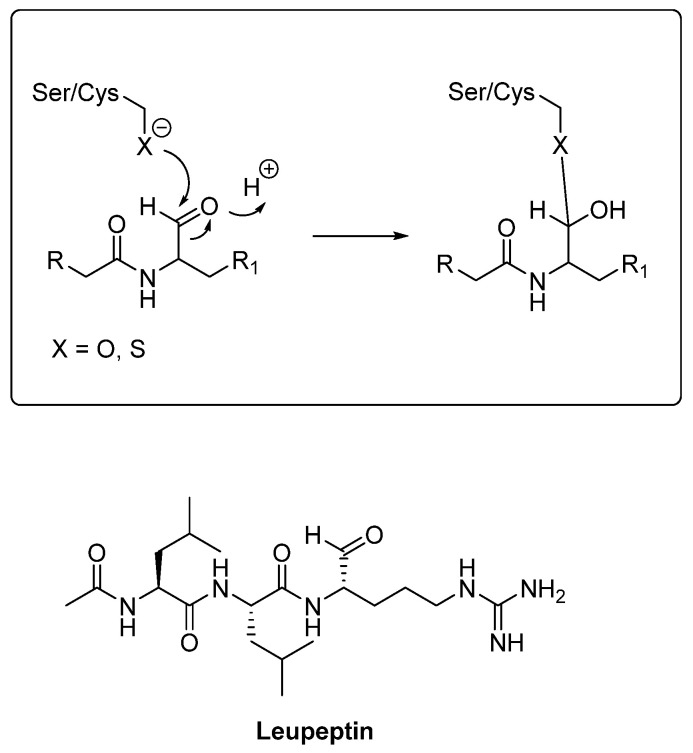
Schematic mechanism of inhibition performed by aldehyde substrates and the structure of leupeptin.

One of the first natural products isolated as a cathepsin inhibitor was leupeptin (N-acetyl-*L*-leucyl-*L*-leucyl-*L*-argininal, Figure 8), identified in 1969 from a strain of *Streptomyces exfoliatus*. Leupeptin is a peptide aldehyde that exhibits inhibitory activity against cathepsins A and B. Cysteine and serine proteases make a nucleophilic attack on the aldehyde group of leupeptin, as shown in Figure 8.

**Figure 9:**
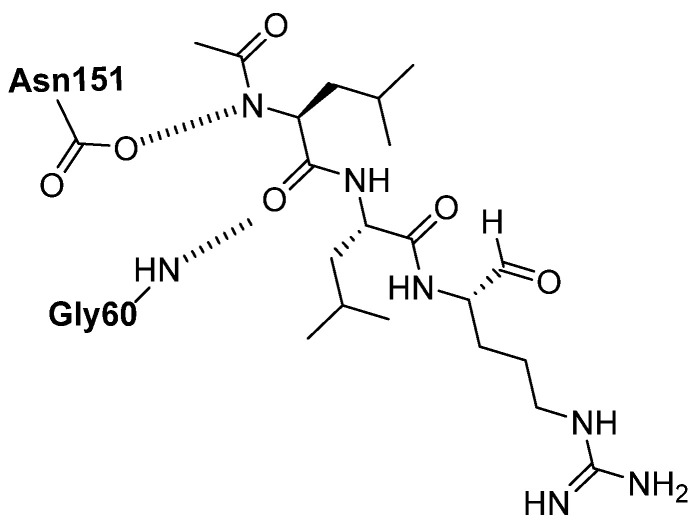
Cathepsin B-leupeptin complex and binding. Adapted from Khara et al. [74].

Binding studies performed on the cathepsin B-leupeptin complex showed a hydrogen bond between the nitrogen of Gly60 of the enzyme with the oxygen 10 of leupeptin, and between asparagine 151 of cathepsin B and the nitrogen atom 3 of the leupeptin ligand (Figure 9) [74].

**Figure 10:**
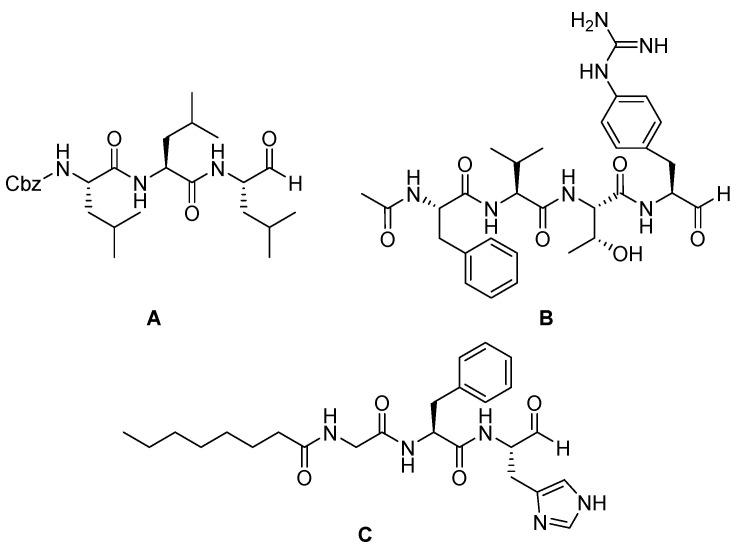
The most potent peptide aldehyde developed by Bartholomew et al. (**A**) [75], Lesner et al. (**B**) [76] and Di Micco et al. (**C**) [77].

Over the years, several other aldehyde inhibitors have been developed. For instance, Bartholomew et al. synthesized a small library of peptide aldehyde inhibitors active towards cathepsin K, finding Cbz-Leu-Leu-Leu-H as the most potent of the series (in vitro enzyme inhibition K_i_ = 1.4 nM) (Figure 10A) [75].

Also, Lesner et al. designed and synthesized a series of small peptide aldehydes as cathepsin G inhibitors, with a general structure Ac-Phe-Val-Thr-X-CHO (where X could be Arg, Lys, Phe, Tyr, p-nitro-*L*-phenylalanine (Nif), pyridyl-*L*-alanine (Pal), 4-amino-*L*-phenylalanine (Amf), and p-guanidine-*L*-phenylalanine (Gnf)). From this study, Ac-Phe-Val-Thr-Gnf-CHO exhibits a K_i_ = 22 nM over CTSG (Figure 10B) [76].

More recently, Di Micco and coworkers [77] described the development of new tripeptide derivatives against SARS-CoV-2 Mpro. Mpro is a cysteine protease that plays a pivotal role in viral replication [78]. The most active compound of the series (58, Octanoyl-Gly-Phe-His-CHO) (Figure 10C) forms a covalent bond between the aldehyde carbon and Cys145 of Mpro, in accordance with the inhibition mechanism of cysteine proteases.

All this evidence confirms that the peptide aldehyde is a suitable scaffold for inhibiting cysteine and serine proteases.

- **Epoxysuccinate inhibitors**

Among the various natural cathepsin inhibitors, E-64, derived from the fungus Aspergillus japonicus, is one of the most widely used in pharmacological research due to its greater selectivity, low toxicity, and strong potency, particularly against CTSB and CTSL. E64 showed an IC_50_ of 4.7 nM against CTSB (measured under standardized fluorescence-based conditions) and 2.5 nM against CTSL [78,79].

**Figure 11:**
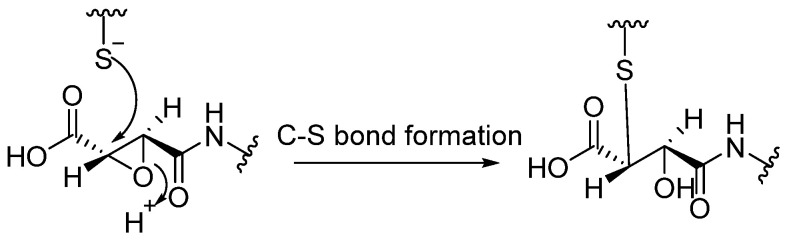
The general mechanism of inhibition by epoxy-inhibitors. Adapted from Liu et al. [80].

E-64 (*L*-trans-Epoxysuccinyl-leucylamido(4-guanidino)butane) represents the lead of the epoxysuccinates class of cathepsin inhibitors. The epoxide group is responsible for the inhibition mechanism; in fact, it participates in the covalent bonding of the catalytic cysteine, inactivating the enzyme (Figure 11).

**Figure 12:**
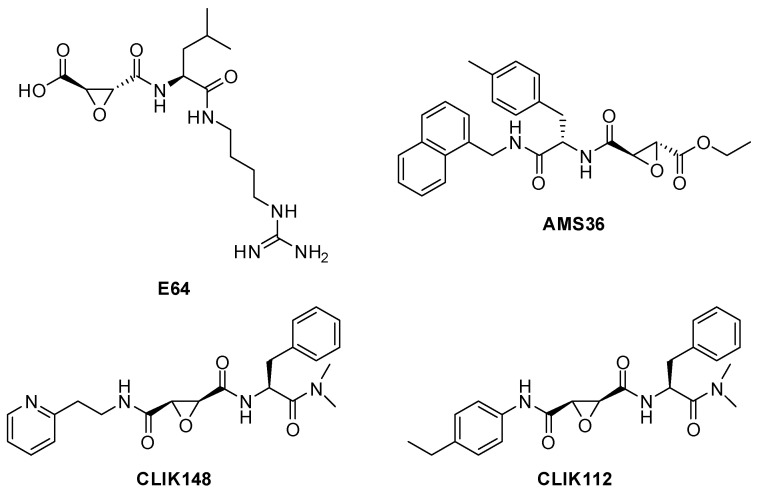
Structure of some epoxysuccinate-based inhibitors.

Starting from E64, Katanuma et al. developed several analogues, named CLIK (Cathepsin L Inhibitor by Katunuma). All these analogues share a common chemical moiety, N-(trans-carbamoyloxyrane-2-carbonyl)-*L*-phenylalanine-dimethylamide. This moiety is essential to form a thioether specifically with the active site cysteine-SH of cathepsin L. The three-dimensional arrangement of the molecules is also important for activity; in fact, CLIK-112, which has an S-S stereostructure specific for epoxysuccinate rings, showed potent inhibition of cathepsin L, whereas its R-R analogue, CLIK-141, showed no inhibition. This indicates that the S-S stereostructure is important for the inhibition of cathepsin L. In the same study, Katanuma et al. demonstrated that the specificity towards cathepsin L is thus achieved by the rigidity and bulkiness of the phenyl ring attached directly to the amino group of the common fragment (Figure 12) [81].

In the same study, another synthesized derivative, CLIK-148, emerged as one of the most stable and selective compounds of the series (Figure 12).

Belonging to the class of epoxysuccinates, AMS36 contains a non-natural p-methylphenylalanine, which drives the selectivity towards cathepsin X (Figure 12) [82].

E-64d ((2S,3S)-trans-epoxysuccinyl-*L*-leucylamido-3-methylbutane ethyl ester) is the ethyl ester prodrug of its active acid form, E-64c, making it structurally analogous to E-64 but with enhanced cell permeability and in vivo availability. Once administered, E-64d is rapidly hydrolyzed to E-64c, which irreversibly inhibits cysteine proteases by covalently binding to their active-site thiol groups, in the same way as its parent compound E-64.

**Figure 13:**
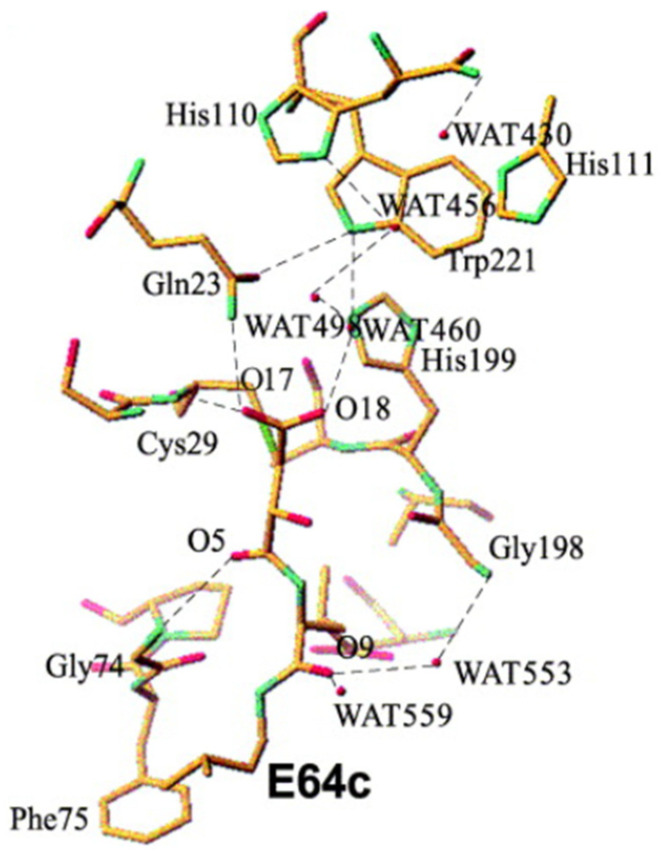
Interactions between E64c and cathepsin B. Adapted from Yamamoto et al. [83].

The crystal structure of E64c and cathepsin B was resolved in 2002 by Yamamoto et al. [83]. As shown in Figure 13, direct hydrogen bonds between E64c and cathepsin B are formed between O5-Gly74NH, O17-Gln23Nε2H and O17-Cys29NH. The latter two hydrogen bonds play an important role in the catalytic reaction [84]. Electrostatic and hydrophobic interactions form between the inhibitor and the enzyme, in particular: N10-Gly74O, C15-Phe75Cζ, C22-Pro76Cδ, C21-Pro76Cγ, C21-Gly198Cα, C21-Ala173Cβ, C21-Ala200Cβ, C11-Phe75Cδ2, and C12-Phe75Cε2. All these bonds form the interactions named S2-P2 and S3-P3. The Leucine residue of E64c is located at the hydrophobic S2 pocket of the protein, which is formed by Pro76, Ala173, Gly198 and Ala200. Also, the methylbutyl moiety of E64c makes a hydrophobic interaction with Phe75 [83].

**Figure 14:**
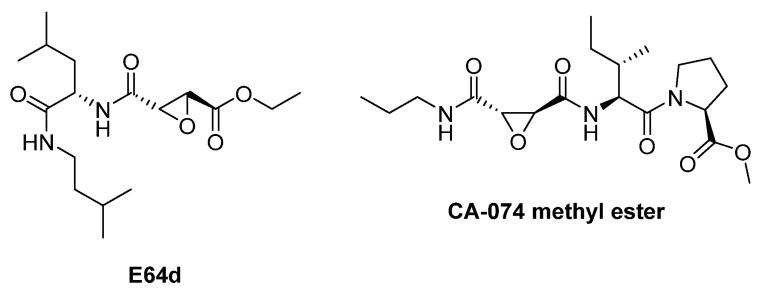
Structures of E64d and CA-074 methyl ester.

CA-074Me (L-3-trans-(Propylcarbamoyl)oxirane-2-carbonyl]-*L*-isoleucyl-*L*-proline methyl ester, 2.24 nM against cathepsin B), and its acid form CA-074 are another CTSB inhibitors that contain the epoxy group (Figure 14).

- **Diazomethylketone inhibitors**

**Figure 15:**
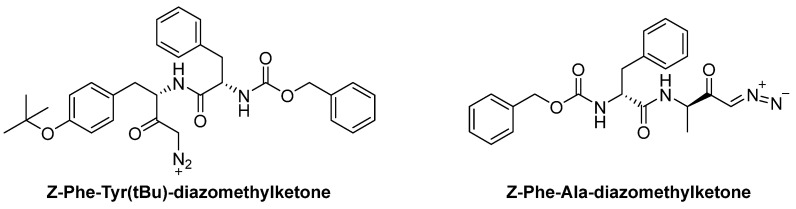
Structures of Z-FY(t-Bu)-DMK and Z-Phe-Ala-diazomethylketone.

This class of compounds acts as inhibitors of cysteine and serine proteases. Some members of this class of inhibitors are Z-FY(t-Bu)-DMK and Z-Phe-Ala-diazomethylketone (PADK, Figure 15). PADK is currently under investigation as a potential treatment for Alzheimer’s disease, due to its ability to disrupt and remodel the early oligomerization of Aβ1-42 [85]. Both of them act as irreversible inhibitors of CTSL. The chemical structure of this class of compounds may be divided into two parts; the peptidic one interacts with the binding pocket of the cathepsin L, and this part contains a benzyloxy carbonyl group as a protective group. The second part of the molecule is characterized by the reactive group that interacts with the CTSL active site; this group is a diazomethylketone, which has been shown to be effective against lysosomal cysteine proteases due to its ability to penetrate cells of various types [86].

**Figure 16:**
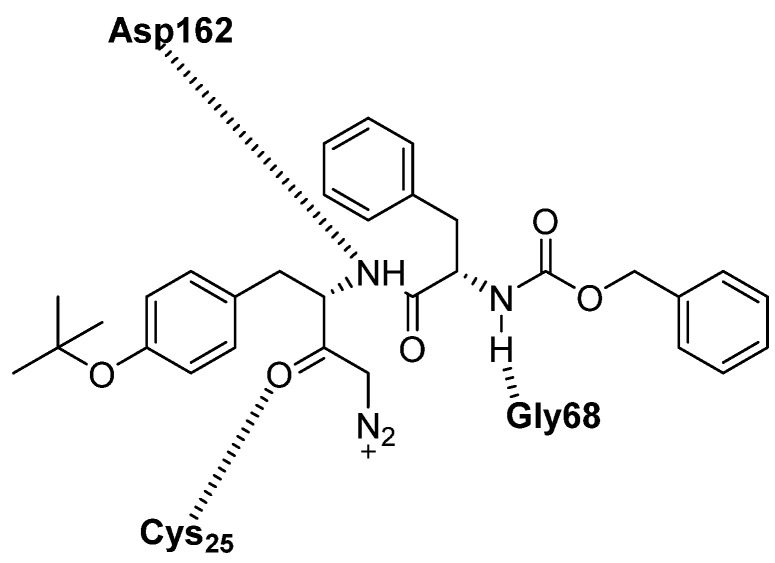
The primary interaction between Z-FY(t-Bu)-DMK and cathepsin L. Adapted from Rajesh T. Shenoy and J. Sivaraman [86].

The structure of Z-FY(t-Bu)-DMK complex with cathepsin L has been solved. Cys25 in the active site of CTSL is covalently bound to the molecule by a thioester bond. In the peptidic part of the inhibitor, the phenylalanine and the Z group occupy S1, S2, and S3 pockets of the protein. In the S1 pocket, the backbone amide of Tyr(t-Bu) group forms a hydrogen bond with the carbonyl oxygen of Asp162. In addition, in the S3 site, there are two hydrogen bonds between the backbones of the inhibitor phenyl group and the Gly68 of CTSL (Figure 16).

## 4. Targeting Cathepsins as a Therapeutic Approach

The role of cathepsins has been widely studied in several pathological conditions, particularly in neurodegenerative diseases, cancer, and viral pathogenesis, as well as in obesity, cardiovascular diseases, and others [15]. Several neurodegenerative diseases, such as Parkinson’s and Alzheimer’s diseases, are characterized by neuroinflammation, which, in the early steps, acts to protect the central nervous system, for instance, removing toxic aggregates [87]. However, chronic inflammation and exposure to cytokines and other inflammatory mediators have detrimental consequences on neuronal cells [88].

Given that cathepsins are key players in neuroinflammation, as previously described, various inhibitors, both synthetic and natural, have been proposed as potential agents for the treatment of neurodegenerative disorders. Some beneficial effects have been demonstrated for cystatins, a superfamily of cysteine cathepsin inhibitors. However, due to their limited selectivity, numerous side effects are anticipated when these inhibitors are used as drugs for patient treatment.

Another interesting therapeutic approach is enzyme replacement therapy (ERT), which aims to provide recombinant enzymes to enhance lysosomal activity and facilitate the clearance of accumulated substrates [89], thereby improving symptoms in various lysosomal storage diseases. This approach leverages the cells’ ability to endocytose intravenously administered enzymes, which are then delivered to the lysosomes. However, a significant limitation of ERT is the blood–brain barrier, which prevents enzymes from reaching the brain. Preclinical studies in animal models with CTSD deficiency have shown that intracerebral infusion of recombinant proCTSD reduces accumulated substrates, reduces neuroinflammation, and, in some cases, improves the overall phenotype of the mice. Similar applications with proCTSL and proCTSB reduced specific substrates and astrogliosis, although without effects on mortality or weight. More recently, recombinant proCTSD has been used in dopaminergic neurons derived from patients with Parkinson’s disease, demonstrating reduced pathological α-synuclein aggregation and improvements in animal models with CTSD deficiency.

Although most studies focus on protease inhibition, it is conceivable that cathepsin activity could also be enhanced through indirect mechanisms, such as lysosomal acidification, TFEB/TFE3-mediated biogenesis, autophagic flux, or modulation of endogenous inhibitors. While direct evidence is limited, this hypothesis offers a complementary perspective on cathepsin regulation. Given the growing number of studies on the cellular mechanisms underlying these phenomena, we found it interesting to review the latest discoveries with a primary focus on cathepsin inhibition. In this connection, the following section provides an in-depth analysis of the underlying mechanisms.

### 4.1. Role of Cathepsins in Parkinson’s Disease and Possible Therapeutic Approaches

Parkinson’s disease (PD) is a progressive neurodegenerative disorder characterized by the selective loss of dopaminergic neurons in the substantia nigra pars compacta and the accumulation of misfolded α-synuclein (αSyn), which aggregates into Lewy bodies and Lewy neurites, key pathological hallmarks of the disease [90]. The clinical manifestations include resting tremor, rigidity, bradykinesia, and postural instability. Although the precise etiology remains elusive, both genetic and environmental factors have been implicated in its pathogenesis [91]. In recent years, increasing evidence has highlighted the critical role of impaired protein clearance pathways, particularly the autophagy-lysosomal system, in PD development [92]. Lysosomal dysfunction impairs the degradation of damaged proteins and organelles, contributing to αSyn aggregation and neuronal toxicity [93]. Among lysosomal proteases, cathepsins, especially CTSD, CTSB, CTSL, and CTSX, have been identified as essential enzymes for maintaining proteostasis and modulating neuroinflammation [94,95]. Experimental models have demonstrated that CTSD deficiency promotes αSyn accumulation and neurodegeneration, whereas supplementation with CTSD can exert neuroprotective effects [96,97]. Moreover, mutations in genes such as GBA, LRRK2, and ATP13A2, which are associated with familial or sporadic PD, have been shown to impair lysosomal function and reduce cathepsin activity, thereby promoting αSyn pathology [35]. These findings support the view that lysosomal dysfunction and cathepsin dysregulation are central mechanisms in PD pathogenesis and represent potential therapeutic targets.

Research has identified autophagy-related components, including CTSD and CTSB, as well as neuroinflammation, as significant risk factors for PD. These findings suggest that lysosomal dysfunction may play a crucial role in the pathology associated with α-synuclein aggregation [40,96]. Specifically, studies in CTSD knockout mice have shown a marked neuronal accumulation of αSyn, while CTSD haploinsufficiency has been linked to diminished lysosomal activity and enhanced propagation of αSyn aggregates. Intracranial injection of recombinant human proCTSD in these mice elevated lysosomal CTSD levels and reduced pathological αSyn conformers, thereby conferring neuroprotective effects [40]. This highlights the critical contribution of lysosomal impairment to the progression of αSyn-related neurodegeneration in PD.

In vitro experiments show that CTSD mediates αSyn degradation, forming C-terminally truncated species. Knockdown of CTSD in αSyn-overexpressing cells increased total αSyn levels by 28% and doubled lysosomal αSyn levels, suggesting CTSD’s crucial role in preventing toxic αSyn aggregate formation through proteolysis. However, CTSD alone cannot fully degrade αSyn, only cleaving specific peptide bonds (e.g., Phe4/Met5, Phe94/Val95), resulting in the formation of amyloidogenic fragments (1–94, 5–94, and 95–140), indicating the need for additional proteases or environmental factors for complete degradation [39].

CTSB and CTSL have also been implicated in the degradation of αSyn. These cathepsins target different regions of αSyn: CTSB cleaves sites such as Ala90/Ala91 and Gly14/Val15, and CTSL cleaves sites such as Met5/Lys6 and Asn103/Glu104. Among these, CTSL has shown the highest efficiency in degrading αSyn, followed by CTSB and CTSD [98]. The importance of CTSB and CTSL in αSyn degradation is further underscored by findings related to the G2019S mutation in leucine-rich repeat kinase 2 (LRRK2), the most common genetic cause of PD. This mutation impairs lysosomal function, reducing the activity of CTSB and CTSL and thereby promoting the aggregation of αSyn [99]. Despite this, the exact mechanism by which LRRK2 G2019S inhibits lysosomal function remains unclear, although studies suggest it might also inhibit chaperone-mediated autophagy [100].

In addition to their role in αSyn degradation, cathepsins are also involved in neuroinflammation, a hallmark of PD pathophysiology. The release of CTSB from lysosomes has been shown to activate the NLRP3 inflammasome, leading to the production of pro-inflammatory cytokines such as IL-1β. This process is exacerbated in the absence of ATP13A2, a lysosomal P5-type transmembrane ATPase, leading to increased lysosomal membrane permeabilization and further CTSB release. Freeman et al. found that after neuronal cell lines take up α-synuclein aggregates via endocytosis, these aggregates can cause lysosomal rupture and trigger an increase in ROS, which is dependent on cathepsin B [30]. The activation of the NLRP3 inflammasome by CTSB has been linked to the progression of neuroinflammation and neuronal degeneration in PD. In PD models of primary neurons and astrocytes, the absence of ATP13A2 increases CTSB release, activating the NLRP3 inflammasome, which links neuroinflammation to lysosomal dysfunction in astrocytes [101].

Moreover, other cathepsins, such as CTSX, have been identified as potential therapeutic targets in PD. CTSX upregulation has been observed in PD models, contributing to the progressive loss of dopaminergic neurons. Inhibiting CTSX expression or activity may offer neuroprotective benefits by reducing neuroinflammation and preserving the nigrostriatal dopaminergic system [102]. Particularly, cathepsin X plays crucial roles in 6-hydroxydopamine (6-OHDA)-induced neuroinflammation and neuronal death [103]. In hemiparkinsonian rats, excitotoxicity induced by 6-OHDA revealed that CTSX overexpression persisted for more than 4 weeks, resulting in the complete loss of dopaminergic neurons. These findings suggest that CTSX upregulation in the damaged dopaminergic system may be a pathogenic factor in PD, and targeting CTSX expression or activity could be a promising therapeutic strategy to protect the nigrostriatal dopaminergic pathway. Furthermore, the nuclear translocation of NF-κB was reduced by the cathepsin X inhibitor AMS36, which blocked the degradation of the Iκb and caspase 3 cleavage in 6-OHDA-treated PC12 cells. Similarly, the downregulation of cathepsin X expression by siRNA in the same cells attenuated 6-OHDA-induced neuronal death, indicating that cathepsin X is directly involved in neuroinflammation and the progression of neurodegenerative diseases.

**Figure 17:**
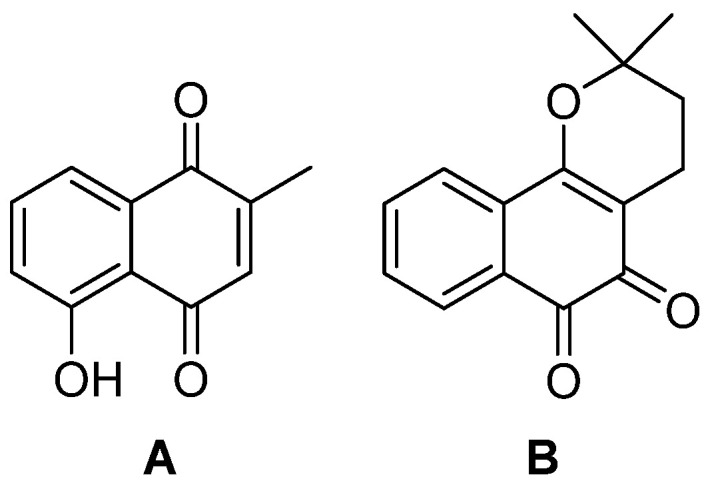
Plumbagin (**A**) and β-lapachone (**B**) structures.

Another study, using artificial intelligence, identified new CTSL inhibitors, of which 13 molecules showed 90% inhibition of CTSL with dose-dependent effects, including plumbagin and β-lapachone (Figure 17), which are promising drug candidates, even if they are currently under investigation for their anticancer and antiproliferative properties [104,105].

In vitro neuroprotection by a cathepsin L inhibitor has recently been demonstrated in a SH-SY5Y cell model of PD, where an irreversible cathepsin L inhibitor, Z-FY(t-Bu)-DMK, significantly reduces 6-OHDA-induced apoptosis [33].

The involvement of cathepsins in PD is further complicated by their interaction with other genetic factors, such as the glucocerebrosidase β1 (GBA) gene. Variants in the GBA gene, which encodes a lysosomal enzyme, have been associated with reduced cathepsin activity and an increased risk of PD. The loss of GBA function leads to lysosomal dysfunction, impairing the degradation of αSyn and contributing to its accumulation in neurons [106].

### 4.2. Role of Cathepsins in Alzheimer’s Disease and Possible Therapeutic Approaches

Alzheimer’s disease (AD) is a progressive neurodegenerative disorder and the leading cause of dementia worldwide, accounting for 60–70% of cases. Clinically, it is characterized by a gradual decline in memory, cognitive function, and behavioral abilities, ultimately resulting in complete functional dependence and death. Neuropathologically, AD is defined by the extracellular accumulation of amyloid-β (Aβ) plaques and the intracellular aggregation of hyperphosphorylated tau protein forming neurofibrillary tangles, particularly within the hippocampus and cerebral cortex. These pathological hallmarks are accompanied by widespread synaptic dysfunction, neuroinflammation, and neuronal loss [107,108].

While the amyloid cascade hypothesis has long dominated the field, proposing that Aβ deposition initiates a series of downstream pathological events, this model fails to account for the complexity and heterogeneity of AD pathogenesis fully. In recent years, growing attention has turned toward lysosomal dysfunction, impaired proteostasis, and the dysregulation of proteolytic enzymes, particularly cysteine cathepsins, as critical contributors to disease onset and progression [109,110].

Among these, cathepsins B, X, L, and D have emerged as pivotal players due to their multifaceted roles in protein turnover, APP and Aβ processing, and modulation of neuroinflammatory and apoptotic pathways. These enzymes are primarily localized to the lysosomal compartment, where they participate in the degradation of long-lived proteins and organelles via autophagy; however, under pathological conditions, their aberrant activity or mislocalization may exacerbate neurodegeneration [111].

A central focus of AD research has been on the role of amyloid-beta (Aβ) peptides in disease progression. The “amyloid cascade hypothesis” posits that the accumulation of Aβ peptides is the primary cause of AD, triggering a cascade of events that includes tau pathology, synaptic dysfunction, and neuronal death. However, this hypothesis alone has not fully explained all aspects of AD, leading researchers to explore additional mechanisms, including lysosomal dysfunction and the role of cysteine cathepsins in the disease [112].

Cysteine cathepsins, particularly cathepsins B, X, and L, have emerged as significant players in the pathology of AD. These proteases are involved in various cellular processes, including protein degradation, inflammation, and apoptosis. CTSB has been of particular interest due to its dual role in AD. On one hand, CTSB exhibits β-secretase activity, contributing to the generation of Aβ peptides by cleaving the amyloid precursor protein (APP). Deletion of the CTSB gene in mice expressing human wild-type amyloid precursor protein (WT APP) has been shown to significantly impact AD pathology, resulting in a 67% reduction in cerebral levels of Aβ-40 and Aβ-42, as well as a decrease in the β-secretase-derived C-terminal fragment of APP (CTFβ). This finding suggests that CTSB plays a role in Aβ peptide generation, contributing to amyloid plaque formation [113]. However, research presents a more complex picture of CTSB’s role in AD. Other studies indicate that CTSB also has a neuroprotective function by degrading Aβ peptides, thereby mitigating plaque formation and its associated neurotoxic effects.

For example, Mueller-Steiner et al. demonstrated that inhibiting CTSB in mice expressing a familial mutant form of APP (hAPP) led to increased Aβ1-42 deposition and exacerbated amyloid plaque accumulation. This effect was also observed in vitro when synthetic Aβ1-42 was incubated with purified CTSB at pH 6, mimicking the acidic environment of endosomes, where CTSB likely encounters intracellular Aβ [114]. The interaction between Aβ1-42 and CTSB led to the proteolytic cleavage of Aβ1-42 into shorter fragments, including Aβ1-40, Aβ1-38, and Aβ1-33, indicating that CTSB can degrade Aβ1-42 into less pathogenic forms. The specific truncations were confirmed to be dependent on CTSB’s proteolytic activity, as they were not observed in the presence of the CTSB inhibitor CA-074 or in the absence of CTSB.

Further studies have supported CTSB’s dual role [115]. In vitro experiments have shown that both CTSB and CTSL are involved in the degradation of Aβ peptides and C-terminal fragments of APP. This was further validated by assessing the effects of inhibiting or knocking down CTSB and CTSL on the levels of key Alzheimer’s-related proteins, including Aβ, APP-CTF, and BACE1. In Chinese hamster ovary cells (CHOwt), pharmacological inhibition of CTSB and CTSL using PADK (Z-Phe-Ala-diazomethylketone) led to a significant accumulation of BACE1 and APP-CTF. Similar results were observed in human neuroblastoma cells SH-SY5Y, indicating that the inhibition of these cathepsins disrupts the normal degradation pathway of these proteins.

Additionally, studies using CTSB and CTSL knockout mouse embryonic fibroblasts (MEFs) revealed a substantial accumulation of BACE1 and APP-CTF in double-knockout cells. In contrast, the levels of full-length APP (fl-APP) remained unchanged. This suggests that CTSB and CTSL are crucial for the lysosomal degradation of BACE1 and APP-CTF. Interestingly, single knockout studies indicated that CTSL may be the primary cathepsin involved in this degradation process [115].

In vivo studies further highlight CTSB’s neuroprotective potential [116]. Increased neuronal CTSB activity has been linked to reduced Aβ formation from WT APP. Moreover, silencing cystatin B, an endogenous inhibitor of cysteine proteases, in AD mouse models reduced intraneuronal accumulation of Aβ40 and Aβ42 and improved behavioral outcomes. This effect was associated with enhanced lysosomal enzyme activity, including that of CTSB, underscoring the potential therapeutic role of modulating cathepsin activity in AD.

Indeed, it was found that Aß levels are significantly reduced in vivo by the cysteine protease inhibitor E64d and the related inhibitor CA-074Me, which specifically inhibits intracellular cathepsin B.

However, the results are controversial, as is the role of specific cathepsins. Cysteine protease inhibitors, particularly cystatin C, may impair cathepsin B ß-secretase activity and the release of Aß peptide in transgenic AD mice. This aligns with findings that lower levels of cystatin C are associated with a higher risk of AD [117]. Indeed, many studies demonstrate that cystatin C (CysC) is a major endogenous inhibitor of cysteine proteases, including CTSB, thereby blocking Aß degradation by CTSB. In particular, Sun et al. demonstrated that reducing CysC activity increases CTSB activity and lowers soluble Aß levels. This was shown in mixed primary cortical cultures of neurons and glia, where cultures, with or without CysC, were infected with an adenoviral vector encoding the hAPP cDNA. Aβ1-42 levels were significantly lower in the supernatants of cultures without the CysC inhibitor, consistent with increased CTSB activity [117].

Other modulators of CTSB include natural compounds derived from marine organisms, such as cyanobacteria, rhodophytes, sponges, mollusks, and phaeophytes, which are often used as semisynthetic precursors. In a study by Phan et al., specific tests were conducted to monitor the proteolytic activity of these compounds, where each molecule was pre-incubated with CTSB at pH 7.2 and pH 4.6.

**Figure 18:**
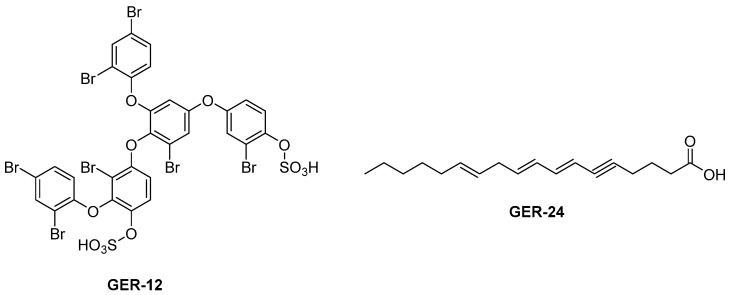
Structures of GER-12 and GER-24.

This study underscores the importance of using specific biological pH conditions to screen for natural modulators of cathepsin B, given that it performs different functions depending on the pH at which it is found: acidic (in lysosomes) or neutral (in the cytoplasm and other extralysosomal compartments). Research has identified natural compounds that selectively inhibit cathepsin B at pH 4.6 or 7.2, showing how pH influences the enzyme’s specificity towards substrates and inhibitors; for example, at acidic pH, cathepsin B prefers glutamic acid (Glu) residues, whereas this preference disappears at neutral pH. In fact, natural products such as GER-12 (crossbyanol B, from cyanobacteria) and GER-24 ((7Z,9Z,12Z)-octadeca-7,9,12-trien-5-ynoic acid, from Rhodophyta) (Figure 18), containing a group that mimics Glu, have shown specific modulation activity at acidic pH. This opens up the possibility of developing selective inhibitors targeting the various functions of cathepsin B [118].

The cysteine protease inhibitor E64d, previously mentioned, is among the top candidates for new therapeutic strategies. In fact, E64d has been found to improve memory and reduce amyloid plaques in the brain, a hallmark of Alzheimer’s disease.

A significant result of this study is that administering E64d in the diet of AβPPL on transgenic mice, which model Alzheimer’s disease, significantly improved memory deficits and retention. Notably, even older mice with established memory problems showed improvements after E64d treatment, indicating that E64d can slow or halt memory decline once it has manifested.

The study also found that E64d reduces brain Aβ levels by inhibiting cathepsin B, without affecting BACE1, an enzyme involved not only in Aβ production but also in synaptic maintenance and myelination. This is important because it shows that E64d reduces brain Aβ by specifically inhibiting cathepsin B, a key enzyme in Aβ production. Additionally, E64d has been shown to increase sAPPα levels, a protein known for its neuroprotective effects, further suggesting that E64d can protect neurons and improve cognitive function.

Finally, despite inhibiting several proteases, E64d has been proven safe in humans during previous clinical trials for other diseases, such as muscular dystrophy, showing good bioavailability and a wide therapeutic window.

CTSX is another protease implicated in AD, particularly in its role in neuroinflammation and plaque formation. CTSX is associated with senile plaques in both AD patients and transgenic mouse models of AD, such as APP/PS1 [119] and Tg2576 [120]. CTSX regulates γ-enolase activity, a neurotrophic factor that promotes neuronal survival, by cleaving its C-terminal domain. This proteolytic activity of CTSX may contribute to the neurodegenerative processes observed in AD by impairing γ-enolase’s neuroprotective function. Microscopic examination of brain cryosections revealed upregulated γ-enolase and CTSX mRNA corresponding to senile plaques. The upregulation of γ-enolase as a protective factor in microglial cells in response to Aβ1-42 and Aβ25-35 peptides has been confirmed in the mouse microglial cell line EOC 13.31 and primary microglia. However, this effect was reversed by CTSX’s proteolytic activity. These results demonstrate the upregulation of γ-enolase in microglial cells surrounding amyloid plaques in Tg2576 transgenic mice and highlight its neuroprotective role in Aβ-related neurodegeneration.

Other studies have demonstrated the involvement of myeloid cell cathepsin X in AD, defining it as a potential target for ameliorating mid-to-late-stage disease in APPSWE/PS1 ΔE9 mice [121].

Similarly, the irreversible epoxysuccinyl inhibitor of cathepsin X has been shown to protect against 6-OHDA-induced neurodegeneration. AMS36 was the first irreversible inhibitor based on epoxysuccinyl from the papain family of cysteine proteases to show some degree of specificity towards CatX in mouse tumors. The study by Pišlar et al. shows that exposure to 6-OHDA increases the levels and activity of cathepsin X in undifferentiated PC12 and SH-SY5Y cells. Inhibition of cathepsin X with the irreversible inhibitor AMS36 attenuates the mitochondrial dysfunction and apoptotic neuronal death caused by 6-OHDA. This protective effect is linked to the inhibition of key pro-apoptotic signaling pathways involving caspase-3, Bax, and NF-κB. AMS36 also prevents the reduction in tyrosine hydroxylase (TH) expression, which is crucial for dopamine production, thereby potentially increasing dopamine levels [103].

Moreover, mutations in the CTSD gene are associated with abnormal production of Aβ and tau proteins. Research involving brain samples from AD patients has identified that a specific mutation, the C→T transition in exon 2 of the CTSD gene resulting in the A58V variant, when combined with the apolipoprotein E (APOE) e4 allele, leads to increased accumulation of Aβ deposits [122]. This combination is recognized as a risk factor for both familial and sporadic late-onset AD. However, the A58V variant of CTSD alone does not appear to elevate Aβ plaque levels. This observation aligns with findings from cerebrospinal fluid studies of individuals with this CTSD variant, which showed no significant change in Aβ concentrations but a reduction in tau levels [123]. Further analysis using liquid chromatography-mass spectrometry (LC-MS) confirmed that CTSD can cleave human Aβ1-42, but it does not play a major role in APP processing. This was demonstrated by incubating a recombinant form of human pro-CTSD with Aβ1-42 peptides under acidic conditions and analyzing the resulting fragments [124].

In addition, research by Bai and colleagues has shown that H_2_O_2_-induced oxidative stress leads to overproduction of CTSB. This overexpression of CTSB subsequently activates the NLRP3 inflammasome, underlining its role in neuroinflammation and its potential as a therapeutic target for AD [125]. Specifically, CTSB release triggers NLRP3 inflammasome activation, leading to cleavage of procaspase-1 and increased IL-1β secretion. Elevated IL-1β levels are noted in the cerebrospinal fluid of AD and PD patients [126,127]. Experiments involving BV2 cells treated with H_2_O_2_ showed a marked increase in MDA, a marker of oxidative stress, and a 1.5-fold rise in CTSB expression compared to controls. Additionally, silencing CTSB with shRNA prevented H_2_O_2_-induced NLRP3 activation and inhibited the processing of procaspase-1 into caspase-1. These findings suggest that CTSB plays a key role in H_2_O_2_-induced IL-1β secretion and caspase-1-dependent cell apoptosis.

Recent models of AD propose that lysosomal dysfunction, driven by alterations in cathepsin activity, plays a central role in the disease. Nixon and colleagues have introduced a complex disease model suggesting that the formation of β-amyloid and the functioning of the endolysosomal network (ELN) are crucial for the development and progression of AD. This model posits that dysfunctions within the ELN and β-amyloid generation are closely intertwined, sharing a common genetic foundation. It emphasizes the early roles of endosomes and lysosomes in processing and clearing APP, as well as the detrimental effects of certain APP metabolites on ELN function. Genes associated with β-amyloid production in AD, such as APP, PSEN1/2, and APOE4, directly affect ELN operations. The significance of initial ELN dysfunction in disease development is highlighted by the identification of mutations in over 35 genes related to the ELN that are known to cause familial neurodegenerative disorders. These models emphasize the importance of maintaining lysosomal function and suggest that therapeutic strategies that modulate cathepsin activity could offer benefits in treating or preventing AD [109].

### 4.3. Role of Cathepsins in Huntington’s Disease and Possible Therapeutic Approaches

Huntington’s disease (HD) is an autosomal dominant hereditary neurodegenerative disorder caused by an abnormal expansion of CAG trinucleotide repeats in the HTT gene, which encodes the huntingtin (Htt) protein. This mutation results in a protein with an expanded polyglutamine (polyQ) tract that is prone to misfolding and aggregation, forming toxic intraneuronal inclusions. Clinically, HD is characterized by progressive motor dysfunction, psychiatric symptoms, and cognitive decline, with selective neuronal degeneration most prominent in the striatum [128,129].

Among the pathogenic mechanisms involved, dysfunction of the autophagy-lysosomal system has garnered increasing attention, as the accumulation of misfolded mutant proteins often results from impaired intracellular degradation. In this context, lysosomal cathepsins have emerged as key regulators of mutant huntingtin metabolism and of the neuroinflammatory response associated with the disease [10,130].

Mantle et al. found a notable rise in protease activity, particularly involving cathepsins H and D, in the brain tissue of individuals with HD [131]. On the other hand, CTSL has been shown to completely degrade mutant huntingtin (Htt) in vitro without producing the harmful N-terminal fragments that lead to inclusions and cell death. Furthermore, in brain lysates from HD knock-in mice, N-terminal mutant huntingtin (N-mhtt) fragments accumulated following cathepsin D treatment and decreased when aspartyl protease inhibition was used [41].

However, another study challenges the notion that CTSD is responsible for generating the N-terminal fragments that drive HD progression. Instead, it suggests that CTSD and CTSB might have a neuroprotective role by reducing neuronal death caused by mutated Htt [36]. This is supported by in vitro studies using clonal striatal cells, PC12 cells, and rodent embryonic cells, in which knocking down cathepsin D increased active Htt1-287 or 1-969 levels, decreased cell viability, and increased mutant Htt aggregates when autophagy was blocked. Cells lacking cathepsin D accumulated more N-terminal Htt fragments. In contrast, wild-type cells were more effective in degrading wild-type Htt, underscoring the importance of autophagy in the degradation of N-terminal Htt [132].

Additionally, increased expression of key neuroinflammation mediators, including CCL2 and IL-10, has been observed in the striatum of post-mortem HD patients, suggesting that neuroinflammation, potentially exacerbated by increased cathepsin release, plays a significant role in the progression of neurodegeneration in HD [133].

Some findings suggest that cathepsins L and Z play a protective role in polyQ-related diseases like Huntington’s, where extended polyglutamine (polyQ) sequences in proteins, aggregation-prone polyQ proteins, accumulate in intraneuronal inclusions. Inhibition of cathepsins L and Z using E64d and leupeptin significantly impairs the degradation of polyQ peptides and proteins, leading to the accumulation of toxic aggregates, especially expanded huntingtin exon 1, which correlates with increased cellular toxicity. Cathepsin L is crucial for initiating the breakdown of polyQ sequences within lysosomes, while cathepsin X further processes these fragments. Studies confirm that reducing cathepsin L or X levels in cells or muscle fibers increases the number and size of polyQ aggregates, enhancing their toxic effects, suggesting that they may have a crucial role in host defense against the toxic accumulation of polyQ proteins [134]. Further research is necessary to clarify the role of cathepsins in HD pathogenesis.

### 4.4. Role of Cathepsins in Neuropsychiatric Disorders and Possible Therapeutic Approaches

Psychiatric disorders such as major depressive disorder, bipolar disorder, and schizophrenia represent complex multifactorial conditions characterized by disturbances in mood, cognition, and behavior. These disorders are believed to arise from a combination of genetic, environmental, and neurobiological factors that alter brain function and neural circuitry [135,136]. Neuroinflammation, impaired neuroplasticity, and dysregulated neurotransmitter systems are recognized as key pathophysiological mechanisms contributing to symptomatology and disease progression [137].

Emerging evidence implicates lysosomal proteases, particularly cathepsins, as important modulators in the neurobiological processes underlying psychiatric illnesses. Cathepsins regulate not only intracellular protein turnover but also extracellular signaling pathways, thereby influencing neuronal resilience, synaptic remodeling, and inflammatory responses. Understanding the dual roles of different cathepsins in both protective and pathological processes is critical for developing targeted therapeutic strategies in mental health disorders [138].

Emerging studies correlate the involvement of cathepsins in mental disorders as they play a role in memory function, hyperactivity, depression, and anxiety; thus, altered cathepsin function can lead to psychiatric diseases, such as major depressive illness, bipolar disorder, and schizophrenia [138].

CTSB has been linked to both protective and deleterious effects in the context of anxiety and depression. Transcriptome analysis of inbred mouse lines selected for high or low anxiety-related behaviors revealed that CTSB is associated with reduced anxiety in female mice. This protective effect may be related to CTSB’s role in neuroplasticity, particularly its ability to activate matrix metalloprotease-9 (MMP-9), which is crucial for activity-dependent neuronal remodeling. This suggests that CTSB may enhance neuronal resilience and plasticity, thereby mitigating anxiety and depressive symptoms [139].

However, the role of CTSB in depression is complex. While some studies indicate that CTSB promotes neuroinflammation via activated microglia, which typically exacerbates depression-like behaviors, other findings suggest that high levels of CTSB may protect against these disorders. This dual role could be context-dependent, possibly influenced by the specific conditions under which CTSB is activated or inhibited. Further research is needed to clarify the mechanisms by which CTSB modulates neuroinflammation and its overall impact on mental health [140].

In contrast to CTSB, CTSC appears to play a more uniformly detrimental role in the development of depression and anxiety. Overexpression of CTSC in mice has been shown to exacerbate neuroinflammation and decrease levels of 5-hydroxytryptamine (5-HT), a key neurotransmitter involved in mood regulation. This aligns with the monoamine hypothesis of depression, which posits that reduced levels of monoamines like 5-HT are a fundamental cause of depressive symptoms [141].

Behavioral studies further support the role of CTSC in promoting depression-like behaviors. Mice with CTSC overexpression exhibit heightened anxiety and depression under both acute and chronic stress conditions. Conversely, knockdown of CTSC has been shown to prevent these behaviors, suggesting that CTSC inhibition could be a potential therapeutic strategy for alleviating depression. The neuroinflammatory response driven by CTSC, likely through microglial activation, may be a critical factor in its role in depression [142].

An emerging area of research highlights the role of cathepsins in secretory autophagy, a process by which these enzymes are secreted from cells to regulate extracellular factors. Cathepsins, particularly CTSC, may influence brain-derived neurotrophic factor (BDNF) and the C-terminal fragment of perlecan LG3, both of which are involved in maintaining neuronal homeostasis and plasticity [143]. This extracellular activity of cathepsins adds another layer of complexity to their role in mental disorders, as it suggests that cathepsins can modulate not only intracellular but also extracellular environments, impacting neuronal health in response to stress or trauma. 

**Figure 19:**
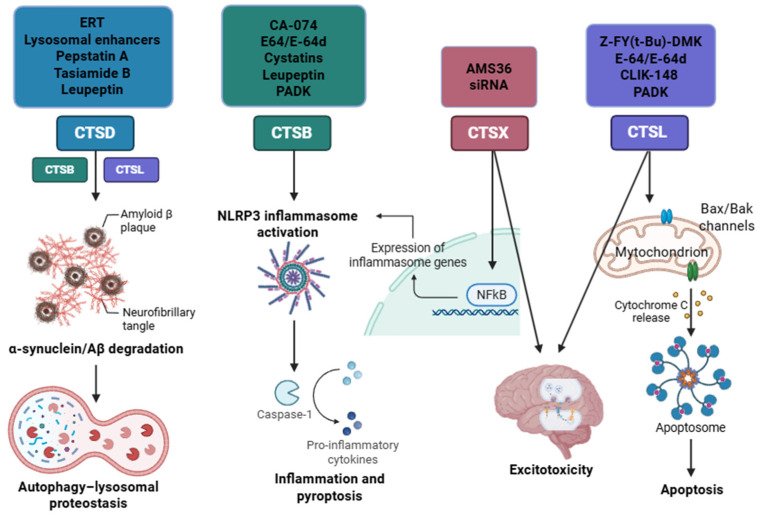
Overview of cathepsin roles and therapeutic modulation in neurodegenerative diseases. The schematic figure illustrates the major lysosomal cathepsins implicated in neurodegeneration: CTSD, CTSB, CTSL, and CTSX, and summarizes their roles in proteostasis, autophagy, neuroinflammation, and neuronal survival. CTSD, the principal lysosomal aspartyl protease, mediates degradation of α-synuclein and APP/Aβ; its deficiency promotes aggregate accumulation, impaired autophagic flux, and neuroinflammation, while enzyme replacement enhances proteostasis. CTSB participates in α-synuclein/Aβ clearance but also contributes to NLRP3 inflammasome activation following lysosomal membrane permeabilization; pharmacological inhibitors (E64, CA-074/CA-074Me) attenuate inflammasome signaling but may reduce proteolytic degradation of aggregates. CTSL efficiently cleaves α-synuclein and regulates autophagy and apoptotic pathways; inhibition (e.g., Z-FY(t-Bu)-DMK) reduces apoptosis and inflammatory signaling, whereas reduced CTSL activity favors protein accumulation. CTSX is upregulated in neurodegenerative conditions and drives NF-κB-dependent neuroinflammation and caspase-mediated apoptosis; selective inhibition (e.g., AMS36) prevents IκB degradation, dampens inflammatory signaling, and protects dopaminergic neurons.

The key mechanisms highlighted and the cathepsin inhibitors discussed in all the text are summarized in the following Figure 19 and Table 2.

## 5. Conclusions

Cathepsins, the principal mediators of lysosomal protein degradation, are intimately linked to the pathogenesis of neurodegenerative disorders, where the accumulation of cell-toxic protein aggregates and inclusions is a defining feature. Their proteolytic activity has the potential to be protective by clearing misfolded and aggregated proteins and alleviating endoplasmic reticulum stress, yet their exact role in disease remains elusive. Emerging evidence suggests that cathepsin activity can act as a double-edged sword: while it contributes to protein quality control and neuronal homeostasis, dysregulated or insufficient activity may exacerbate proteotoxic stress and drive neuronal damage.

Despite compelling preclinical studies, translating cathepsin-targeted strategies into the clinic faces substantial challenges. Many available inhibitors, such as E64d, lack molecular specificity and engage other lysosomal enzymes, while limited brain penetrance and incomplete CNS pharmacokinetic profiling hinder their therapeutic potential. In addition, heterogeneous preclinical models, varying in species, cell lines, and genetic backgrounds, complicate the extrapolation of efficacy to humans.

Indeed, although robust preclinical evidence exists, translating cathepsin-targeting strategies to the clinic remains elusive, mainly due to challenges in achieving isoform selectivity, achieving brain penetration, and ensuring long-term safety. Current insights highlight that manipulating these proteases requires a nuanced approach, balancing inhibition with the restoration of lysosomal competence, rather than simple enzymatic blockade. Moving forward, the development of selective brain-permeable modulators, coupled with rigorous in vivo validation and the integration of pharmacokinetic and biomarker-driven studies, will be essential to bridge the gap between experimental promise and clinical applicability. Future progress will depend on the convergence of medicinal chemistry, molecular neurobiology, and translational pharmacology to transform cathepsin modulation from a preclinical concept into a realistic therapeutic avenue for neurodegenerative and neuropsychiatric disorders.

## Figures and Tables

**Table 1:** Cathepsins’ involvement in the cleavage of protein aggregates and their associated disease.

Enzyme	Substrate	Associated Disease	Role in Neurodegeneration	References
CTSD	APP, Tau, Aβ	AD	Contributes to neurodegeneration by impairing lysosomal acidification, disrupting proteostasis, and promoting the accumulation of misfolded proteins, leading to enhanced neuroinflammatory and degenerative processes	[34]
	αSyn	PD, MSA, DLB		[35]
	HTT	HD		[36]
	PrP	Prion disease		[37]
	ApoE	AD		[38]
CTSB	APP, Aβ	AD	NF-κB activation, mitochondrial transcription factor A degradation, induction of apoptosis via pro-caspase-1/11 activation, and contribution to neurotoxic Aβ production. Also amplifies microglial-driven inflammation and ROS/RNS release.	[34]
	αSyn	PD, MSA, DLB		[39]
	Htt	HD		[36]
	PrP	Prion disease		[37]
CTSL	APP	AD	Facilitates neuroinflammation via activation of caspase-8 and NF-κB pathways, enhances expression of iNOS/COX-2.	[38]
	αSyn	PD, MSA, DLB		[35,39,40]
	Htt	HD		[41]
	PrP	Prion disease		[37]
CTSS	APP, Aβ	AD	Remains active at neutral pH, enabling extracellular matrix degradation and microglial migration.	[42]

**Table 2:** Key cathepsin inhibitors/modulators and their targets.

Modulator/Inhibitor	Class/Functional Group	Target Cathepsin(s)	Mechanism	Uses and References
Pepstatin A	Synthetic peptide, hydroxyethylamine group	CTSD	It acts as a competitive and reversible inhibitor that binds to the active site of aspartyl proteases, blocking their function. It achieves this by mimicking the transition state of the substrate [66].	Classical inhibitor of aspartic proteases; frequently used in autophagy studies [144].
Tasiamide B	Natural peptide, hydroxyethylamine group	CTSD	It acts as a competitive inhibitor, mimicking the tetrahedral intermediate of the enzyme [68].	Hit compound to develop other aspartic inhibitors [67].
TB-9	Synthetic peptide, derivative of tasiamide B	CTSD CTSE	Same mechanism of inhibition of Tasiamide B [67].	Under investigation to improve cell permeability [67,68].
Leupeptin	Peptide aldehyde	CTSA, B, D Reversible inhibitor	It acts as a competitive inhibitor that forms a reversible, covalent hemiacetal bond between its aldehyde group and the enzyme’s active site [69].	Under investigation for Parkinson’s disease [145].
Cbz-Leu-Leu-Leu-H Ac-Phe-Val-Thr-Gnf-CHO Octanoyl-Gly-Phe-His-CHO	Peptide aldehyde	CTSK, CTSG Mpro	Same mechanism as leupeptin [74].	Under investigations [75,76,77].
E-64	Epoxysuccinate (natural origin)	CTSB, CTSL, CTSK, CTSH, CTSS Irreversible inhibitor	The epoxide group forms a covalent bond, inactivating the enzyme [80].	Widely used in research [79,80].
E-64d (Aloxistatin)	epoxysuccinate	CTSB, CTSL (+calpain) Irreversible inhibitor	Same mechanism as E-64 [80].	Cell-permeable derivative of E-64; used in models of Alzheimer’s and traumatic injury [83].
AMS36	epoxysuccinate	Irreversible inhibitor of CTSX	Same mechanism as E-64 [80].	Under investigation for the treatment of Parkinson’s disease [103].
CA-074 methyl ester	epoxysuccinate	Irreversible inhibitor of CTSB	Same mechanism as E-64 [80].	Under investigation in Alzheimer’s disease [114].
CLIK-148	epoxysuccinate	CTSL	Same mechanism as E-64 [80].	Synthetic analogues of E64, several potential medical applications [146].
Z-FY(t-Bu)-DMK	diazomethylketone	CTSL	It acts as irreversible inhibitor of the enzyme [86].	Mainly used in research [85].
PADK	diazomethylketone	CTSB, CTSL	Same mechanism as Z-FY(t-Bu)-DMK [86].	Inhibits CTSB/CTSL; prevents BACE1 and APP-CTF degradation in cells [85].

## Data Availability

No new data were created or analyzed in this study.
